# Burns of the First and Second Degrees

**Published:** 1891-03

**Authors:** 


IN BURNS of the first and second degrees, Nikolski *Deutsches Med. Wochen.* in *Med. News*, Feb. 15th) employs the following mixture as an external application:

℞ Acid tannic.,

Alcohol, aa p. 10.

Ætheris, p. 80. M.

